# BEaTS-β: an open-source electromechanical bioreactor for simulating human cardiac disease conditions

**DOI:** 10.3389/fbioe.2023.1253602

**Published:** 2023-09-15

**Authors:** Hiroki Takaya, Maxime Comtois-Bona, Ana Spasojevic, David Cortes, Fabio Variola, Wenbin Liang, Marc Ruel, Erik J. Suuronen, Emilio I. Alarcon

**Affiliations:** ^1^ Division of Cardiac Surgery, University of Ottawa Heart Institute, Ottawa, ON, Canada; ^2^ Biomedical Mechanical Engineering, University of Ottawa, Ottawa, ON, Canada; ^3^ Department of Mechanical Engineering, University of Ottawa, Ottawa, ON, Canada; ^4^ Cardiac Electrophysiology Laboratory, University of Ottawa Heart Institute, Ottawa, ON, Canada; ^5^ Department of Cellular and Molecular Medicine, University of Ottawa, Ottawa, ON, Canada; ^6^ Biochemistry, Microbiology and Immunology, University of Ottawa, Ottawa, ON, Canada

**Keywords:** cardiac tissue, bioreactor, iPSC cells, cardiac cells, cardiovascular disease

## Abstract

Heart disease remains the leading cause of worldwide mortality. Although the last decades have broadened our understanding of the biology behind the pathologies of heart disease, *ex vivo* systems capable of mimicking disease progression and abnormal heart function using human cells remain elusive. In this contribution, an open-access electromechanical system (BEaTS-β) capable of mimicking the environment of cardiac disease is reported. BEaTS-β was designed using computer-aided modeling to combine tunable electrical stimulation and mechanical deformation of cells cultured on a flexible elastomer. To recapitulate the clinical scenario of a heart attack more closely, in designing BEaTS-β we considered a device capable to operate under hypoxic conditions. We tested human induced pluripotent stem cell-derived cardiomyocytes, fibroblasts, and coronary artery endothelial cells in our simulated myocardial infarction environment. Our results indicate that, under simulated myocardium infarction, there was a decrease in maturation of cardiomyocytes, and reduced survival of fibroblasts and coronary artery endothelial cells. The open access nature of BEaTS-β will allow for other investigators to use this platform to investigate cardiac cell biology or drug therapeutic efficacy *in vitro* under conditions that simulate arrhythmia and/or myocardial infarction.

## 1 Introduction

Cardiovascular diseases remain the leading cause of morbidity worldwide (World Health Organization, 2022). More than 60% of heart-related deaths are due to occlusion of coronary arteries, causing massive and irreversible cell death at the site of infarction. The death of cardiomyocytes, along with the poor regenerative capacity of the myocardium, results in the formation of a scar that does not exhibit the same physiological characteristics as healthy myocardium. This scar tissue can lead to arrhythmia and reduced heart function that can ultimately result in heart failure (Gaziano et al., 2010). Although interventional and surgical procedures can improve a patient’s prognosis by re-establishing blood flow to the myocardium, restoring cardiac function after myocardial infarction (MI) remains challenging due to the limited ability of the heart to replace the lost cardiomyocytes (Anversa et al., 2002).

In recent decades, advances in tissue engineering and bioprinting have led to increased interest in the development of medical devices and bioreactors to improve the cell culture environment and the function of cultured cells (Khan et al., 2015; Kroll et al., 2017). The *in vitro* culture of cells on conventional 2D substrates and 3D matrices (hydrogels and bioprinted structures) is one of the most important steps in the generation of artificial biomimetic tissues. The generation of these tissues and organs and their nature-mimicking physiology is a multifactorial challenge that includes cell-to-cell signaling, cell-extracellular matrix interactions, and physical cues (mechanical, electrical, chemical) (Khan et al., 2015; Kroll et al., 2017). Advances in the technologies used to generate and regulate these engineered tissues, along with the discovery and use of induced pluripotent stem cells (iPSCs), are opening doors to promising treatments for several human diseases, including cardiovascular diseases (Miklas et al., 2014; Kroll et al., 2017). Although several *in vitro* experiments have been reported on the post-ischemic changes of differentiated cardiomyocytes, these studies only considered environmental conditions such as oxygen level and nutrient deprivation, and incorporate other external factors such as electromechanical stimulation, which is an important factor for cardiomyocyte function (Sacks et al., 2018; Shah et al., 2019; Valimaki et al., 2020). Furthermore, it remains unclear how differentiated cardiac cells interact with non-cardiac myocytes, such as fibroblasts, under physiological or pathological conditions (Rohr, 2012).

Recently, we reported an open-source, 3D-printed electromechanical stimulator that fits into small and regular-sized incubators, minimizes the number of components in the incubator, prevents corrosion and overheating, and reduces overall device cost (Cortes et al., 2020). Electromechanical stimulation with this device was observed to improve the maturation of human iPSC-derived cardiomyocytes (hiPSC-CMs) (Cortes et al., 2020). In this report, we re-engineered the device to make it smaller and suitable to operate at variable rates and pulse durations, such as those observed during myocardial infarction or arrhythmia as well as under hypoxic conditions and evaluated its use for culturing different types of myocardial cells.

## 2 Materials and methods

### 2.1 3D printing of BEaTS-β

**FIGURE 1:**
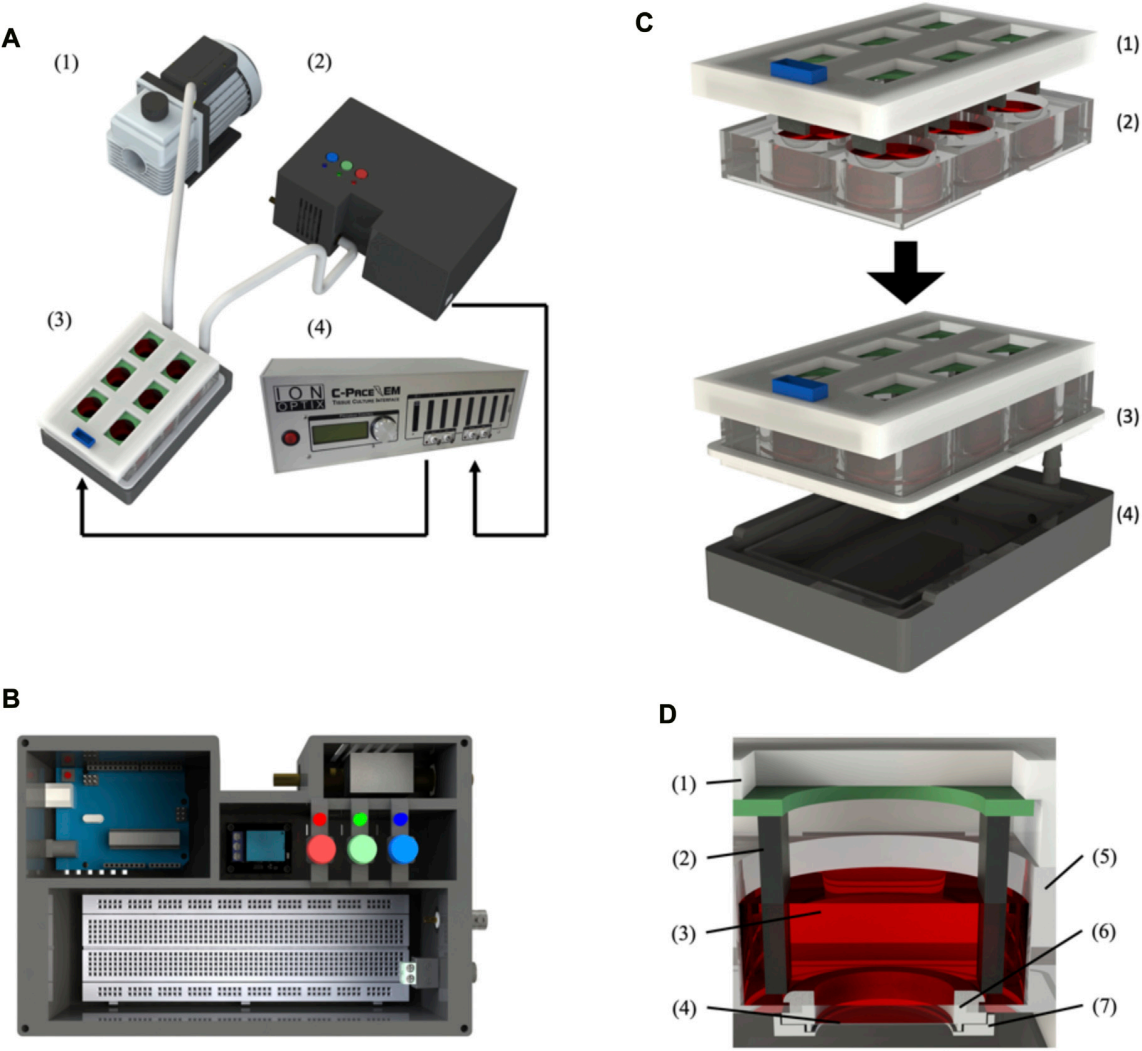
BEaTS-β: a 3D printed device for mechanical and electrical stimulation of cells *in vitro*. **(A)** Assembled BEaTS- β system consisting of (1) a vacuum pump, (2) a modified 6-well culture plate, (3) a control unit containing the Arduino Uno microcontroller and solenoid valve and (4) the C-Pace EP system. **(B)** Interior view of the BEaTS– β control unit. The system contains the Arduino Uno microcontroller, a solenoid valve to regulate the vacuum on the membrane and a BNC port to drive the C-PACE EP system. **(C)** Assembling process for the modified 6-well culture plate containing: (1) the C-Pace EP electrodes, and (2) a 6-well plate with the bottom of the well removed and replaced by flexible, translucent silicone sheets. **(D)** Close-up view of the modified well with (1) the C-Pace EP cover and (2) electrode, (3) Cell media, (4) Flexible membrane, (5) wall of the well plate, (6) Top membrane holder and (7) bottom membrane holder.

All 3D printed parts were made in an Ultimaker S5 (Ultimaker, Netherlands) (Figure 1A and Supporting Material Supplementary Tables S1, S2). The plate holders were printed using Nylon 680 filament (Taulman, United States) as they need to be rigid and autoclavable while the membrane holders were printed using thermoplastic elastomer (TPE 80A) due to the required elasticity of the membrane. Finally, the case of the control unit as well as the rest of the mechanical components were printed using Acrylonitrile butadiene styrene (ABS) filaments for the low cost and ease of printing. Most components of the BEaTS-β device are 3D printed; all. stl files for generating the components of BEaTS-β are available for download at https://figshare.com/s/ce866e8e4312b9cd0ade. The following is a description of the key elements in BEaTS-β.

### 2.2 BEaTS-β assembly

The assembly instructions for the membrane holders and plate seal plate holder, as well as the electrical schematic for the control unit, can be found in the Supplementary Material (Supplementary Figures S1–S3). Briefly, the bottom of a 6-well plate must be removed and replaced by the flexible membranes, which are held in place by the 3D printed membrane holders. Once modified, the 6-well plate is placed on top of the plate holder and sealed using a custom-made silicone seal that is placed around the plate (Figure 1C). Once the plate is fully assembled, the first inlet valve of the plate holder is connected to an air pump while the second inlet valve is connected to a solenoid pump placed outside of the incubator. Finally, the valve is connected to the Arduino board within the control unit. Before each cell experiment, the membranes are washed with 70% ethanol and PBS and subsequently coated with 250 µL of 1X Attachment Factor (ThermoFisher, United States).

### 2.3 Code and stimulation cycles

**FIGURE 2:**
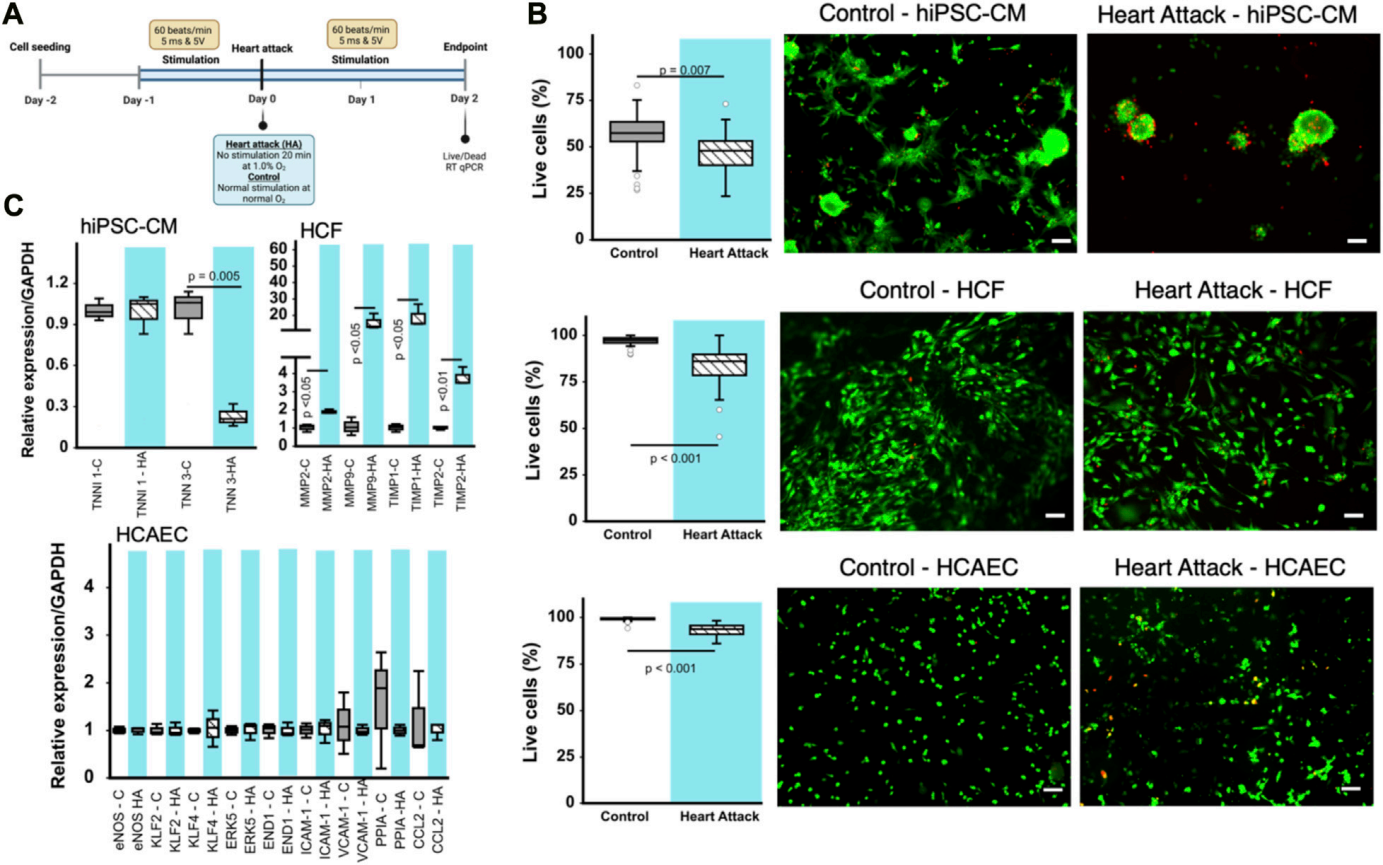
Effect of heart attack simulation on cardiac cell viability and gene expression. **(A)** Schematic for experimental design of *in vitro* cell incubation using BEaTS- β device to mimic heart attack conditions. **(B)** Cell viability measured using Live/Dead kit expressed as percentage of living cells per field of view. Representative images for each type of cell are provided. Scale bars = 100 µm in all cases. **(C)** Relative expression of various genes for the different cell types cultured using BEaTS-β with and without the heart attack simulation protocol. In all cases (n = 3) and *p* values calculated using two-tailed *t*-test.

In BEaTS-β, the code was modified to allow selection from three different stimulation modes with specific BPM (Beats per minutes) (World Health Organization, 2022): normal heartbeat (80 bpm) (Gaziano et al., 2010), arrhythmia (looping 45 bpm, 80 bpm, and 120 bpm), and (Anversa et al., 2002) heart attack (resuming stimulation at 80 bpm after 20 min of electromechanical stimulation discontinuation and another 20 min of recovery at a lowered frequency of 45 bpm, Figure 2A). The electrical schematic for the control unit, can be found in the Supplementary Material (Supplementary Tables S3, S4). After the cells are cultured in well plates and placed in the incubator, the user can select which condition to activate with the press of a button. This choice can be changed in real time, and both mechanical and electrical stimulation are automatically modified based on the user’s choice. Before changing the stimulation cycle, the user must manually change the temperature and gas concentration. In this study, the following cellular experiments were conducted using the heart attack model described in Table 1.

**TABLE 1:** Summary of the different modes of BEaTS-β device. The files contained in Figshare contains all the information for assembling and programming the device to operate the different modes below listed.

Mode		Simulation condition	
		Mechanical beating	Electrical stimulus
Normal heartbeat		80 bpm	5V/cm, 50 m at 80 bpm
Heart Attack	First 20 min (Heart failure)	No Stimulation	No Stimulation
	Next 20 min (recovery)	45 bpm	5V/cm, 50 m at 45 bpm
	Rest of the simulation	80 bpm	No Stimulation
Arrhythmia (Loop)	Normal Heartbeat	80 bpm	5V/cm, 50 m at 80 bpm
	Bradycardia	120 bpm	5V/cm, 50 m at 120 bpm
	Tachycardia	120 bpm	5V/cm, 50 m at 120 bpm

### 2.4 Culture and differentiation of human induced pluripotent stem cells

All of the procedures were approved by the institutional animal care and ethical committee at the University of Ottawa. Investigations involving human tissues conform to the principles outlined in the Declaration of Helsinki, and informed consent was obtained from all subjects prior to their inclusion in the study. The iPSC line was generated from a healthy female volunteer in Dr. Joseph C. Wu’s lab at Stanford Cardiovascular Institute with Institutional Ethics Committee approved protocols (IRB 29904 and SCRO 485) and informed written consent was given prior to the inclusion of the subject in the study. Differentiation of hiPSCs into cardiomyocytes was performed using a 2-D monolayer protocol in a chemically defined medium in Dr. Liang’s laboratory. hiPSCs (passage 20–25) at 80%–90% confluency were treated with 6 µM CHIR99021 (Selleck Chemicals) from day 0–2 in CDM3 medium (RPMI 1640 supplemented with 213 μg/mL L-ascorbic acid2-phosphate and 500 μg/mL recombinant human albumin). Cells were maintained from day 2–4 in CDM3 medium containing 2 µM Wnt-C59 (Selleck Chemicals). At day 4, cells were cultured in control CDM3 medium (without other added factors) and spontaneously beating cells were observed under a microscope at day 6–8. Cells were glucose-starved from day 10–14 to purify cardiomyocytes. Prior to cell seeding in BEaTS-β, 500 µL of Matrigel (Corning, United States of America) was added to each membrane and all membrane plates were incubated at 37°C, 5% CO2 overnight. To lift and reseed hiPSC onto the new membranes, cells were treated with Collagenase B in Ca2+-free Tyrode solution, pH = 6.9 (T6.9). First, 100 mg of Collagenase B was diluted in 1 mL of PBS, aliquoted to 50 µL in 0.6 mL Eppendorf tubes and kept at −80°C. Two aliquoted solutions (100 µL) of the collagenase B/PBS solution were taken out of the −80°C freezer, thawed at room temperature, and diluted in 2 mL of T6.9. The hiPSC-CMs at day 30 of differentiation were washed gently with T6.9. Then, 500 µL of the diluted collagenase B solution was added to each well and the well plates were transferred to the cell incubator at 37°C, 5% CO_2_ and 21% O_2_ for 100 min. After 100 min, the collagenase B solution was quenched by adding 3 mL of cell culture media. Cells were collected in a 15 mL Falcon tube and spun at 300 G for 5 min. Approximately 8.0 × 10^5^ and 1.0 × 10^5^ cells were seeded on each membrane for RT-qPCR and cell viability assay respectively and cultured for 24 h before any stimulation was applied.

### 2.5 Isolation and culture of human cardiac fibroblasts

Human cardiac fibroblasts (HCFs) were isolated from a healthy donor and cultured in DMEM/F-12 (Gibco, United States) containing 10% fetal bovine serum and 1% penicillin/streptomycin. Cells were cultured at 37°C, 5% CO_2_, 21% O_2_. Cells were harvested at 75%–85% confluence to perform the following experiments. To lift and reseed cells onto the new membranes, cells were treated with trypsin-EDTA (0.25%) (Gibco) for 2 min at 37°C, then quenched by adding cell culture media. Cells were collected in a 15 mL Falcon tube and centrifuged at 350 G for 5 min. Approximately 2.0 × 10^5^ and 1.0 × 10^5^ cells were seeded on each membrane for RT-qPCR and cell viability assays, respectively, and cultured for 24 h before any stimulation was applied.

### 2.6 Culture of human coronary artery endothelial cells

Human coronary artery endothelial cells (HCAECs; ATCC, United States of America) were cultured in Endothelial Cell Growth Medium (ATCC) containing 2% FBS and 1% penicillin/streptomycin. Cells were cultured at 37°C, 5% CO_2_, 21% O_2_. Cells were harvested at 75%–85% confluence to perform the following experiments. To lift and reseed cells onto the new membranes, cells were treated with trypsin-EDTA (0.25%) (Gibco) for 5 min at 37°C, then quenched by adding cell culture media. Cells were collected in a 15 mL Falcon tube and centrifuged at 150 G for 5 min. Approximately 4.0 × 10^5^ and 2.0 × 10^5^ cells were seeded on each membrane for RT-qPCR and cell viability assays, respectively, and cultured for 24 h before any stimulation was applied.

### 2.7 *In vitro* heart attack model

This model mimics the myocardial infarction environment, where the heartbeat resumes tens of minutes after a pulseless arrhythmia or cardiac arrest due to a heart attack. The scheme of the experimental protocol is shown in Figure 2A. Each type of cell was incubated for 24 h after seeding onto membrane, the electromechanical stimulation was applied (every 45 m with a duration of 5 m and a voltage of 5 V) for another 24 h. To mimic heart attack conditions *in vitro*, the incubator (SANYO, Japan) was turned to a hypoxic condition saturated with 94% N_2_, 5% CO_2_, and 1% O_2_ for 20 min with interruption of the electromechanical stimulation b BEaTS-β. To mimic reperfusion, the cells were then returned to normoxic culture with 5% CO_2_, 21% O_2_ with resumption of electromechanical stimulation for 48 h and used for further experiments. Resumption to electromechanical stimulation and oxygen concentration was carried out to mimic reestablishment of blood supply post-myocardial infarction. As a control group, cells were seeded onto the membrane, incubated for 24 h, then cultured under normoxia without interruption of electromechanical stimulation for 3 days. Flexible membranes were pre-coated with attachment factor, 500 µL per well (6 h) prior adding cells.

### 2.8 Cell viability assay

Once the electromechanical stimulation protocol was completed, a LIVE/DEAD^®^ Viability/Cytotoxicity Kit (Invitrogen, United States) was used to assess cell viability. Briefly, the medium was removed from the wells and cells were gently washed with phosphate buffer saline (PBS). Then a mixture of 5 mg of calcein AM and 4 mg of ethidium homodimer-1 in 400 mL of PBS was added to each well. The dish was kept in the dark at 37°C for 40 min. The membranes were carefully removed from membrane’s holders and mounted on a new 6-well plate, and then cells were observed using a fluorescence microscope (ZEISS Axio Observer; Zeiss, Germany). Images were taken from 20 random regions. The images were analyzed, and live and dead cells were quantified using the software ImageJ (National Institutes of Health, United States of America).

### 2.9 RNA isolation and qRT-PCR

hiPSC-CMs, fibroblasts and HCAECs were cultured for 3 days after 20-min exposure to hypoxia and no electromechanical stimulation, after which total RNA was isolated using the RNeasy kit (Qiagen). First-strand cDNA was synthesized with Smartscribe Reverse Transcriptase (Takara Bio, United States) and random hexamer primers (Fisher Scientific). Target gene mRNA levels were assessed by quantitative RT-PCR with LightCycler 480 SYBR Green I Mastermix (Roche) and a LightCycler 480 Real-Time PCR System (Roche). The primer sequences used for qRT-PCR are shown in Supplementary Table S5.

RT-PCR was performed under the following conditions: initial activation at 95°C for 5 min followed by 45 cycles of denaturation at 95°C for 15 s, annealing at 60°C for 20 s and 72°C for 20 s. Relative changes in mRNA expression were determined by the ∆∆Ct method, expressed as levels relative to GAPDH. Each sample was run in duplicate.

### 2.10 Statistical analysis

Data are expressed as the mean ± standard error. Statistical analyses were performed using Kaleida Graph 4.5 software (Synergy Software, Reading, PA). All values were analyzed using a two-tailed *t*-test.

## 3 Results and discussion

### 3.1 Mechanical stimulation

The mechanical stimulation of the cells in the device chamber was accomplished by the mechanical stretching of a flexible, translucent silicone membrane substrate (Grace Bio-labs, United States) utilizing a pressure differential generated by a vacuum-driven system (see Figure 1A and methods section for further details). Using a solenoid valve coupled to an Arduino microcontroller, Figure 1B, the opening and closing of the valve can be controlled by the microcontroller such that, when opened, the membranes are at rest and, when closed, a vacuum is applied to the membrane, causing them to deform. The cyclic opening and closing of the valve generate the cyclic strain on the membrane, Figure 1C, which is transmitted to the seeded cells, thus generating a mechanical stimulation like that experienced by the cells in the myocardium (Davis et al., 2015). While the design of the flexible membrane (Figure 1D) remains unchanged from the original device, everything else was updated to reflect the new functionalities of the device as well as improve consistency/reliability. Specifically related to the mechanical stimulation, the 3D printed solenoid valve of the BEaTS-α device was replaced with a magnetic coil-activated solenoid valve, allowing for fast actuation time and increased reliability. The pressure of the vacuum pump can be regulated directly on the vacuum pump and a pressure of 55 kPa was chosen as it produced the desired mechanical deformation of the flexible membrane. As the design of the flexible membrane and the pressure from the vacuum pump remained unchanged compared to the BEaTS-α, the mechanical characterization of the membrane was omitted but specific details about the pressure distribution and deformation of the membrane can be observed in the BEaTS-α article (Cortes et al., 2020). As shown for the BEaTS-α Finite Element Analysis (FEA), the membrane experiences an average strain of 2.34% at normal operating pressure (55 kPa) and the deformation remains homogeneous within an 8 mm radius from the center of the membrane (Cortes et al., 2020).

### 3.2 Electrical stimulation

The electrical stimulation of cells was performed using the commercially available C-PACE EP System. This device uses carbon electrodes arranged in parallel to provide a field of electrical stimulation for the cells. The voltage, frequency, and duration of each pulse can be controlled externally using the device’s interface or by using other compatible devices that can be connected to the system (Figure 1). BEaTS-β uses an Arduino Uno to output a Transistor-Transistor Logic (TTL) signals that control the rate of stimulation applied to the cells with the C-PACE EP system, and the C-PACE EP system uses this TTL signal to controls the periodic stimulation of the cells by outputting a pulsatile electrical signal with a specific voltage, pulse length and frequency. This allows both electrical pacing and mechanical stimulation of the cells to be synchronized, creating a more physiologically relevant model. The BEaTS-β device incorporates a normal heartbeat cycle that delivers an electrical impulse of 5 V/cm with a pulse duration of 50 m every 450 m. In addition to the normal heartbeat cycle present in the BEaTS-α, the device allows for tunable stimulation regimes with the option to select arrhythmia and heart attack cycles (further details are included in Table 1).

The tunable stimulation regime was implanted through the addition of 3 pushbuttons in the newly designed control unit (Figure 1B) to switch between the 3 different operating modes of the device (see Table 1). The control unit also houses the Arduino Uno microcontroller, the main circuit board, the solenoid valve, and a relay to control the solenoid valve.

### 3.3 Culture of different cardiac cell types in the BEaTS-β device

Human iPSC-derived cardiomyocytes (hiPSC-CMs), human cardiac fibroblasts (HCFs), and human coronary artery endothelial cells (HCAECs) were cultured separately in the BEaTS-β device under normal conditions and in conditions that mimic a heart attack (Figure 2A). All cell groups were incubated under normoxia for 24 h followed by electromechanical stimulation for another 24 h. For the heart attack model cells were then exposed to hypoxia for 20 min with no stimulation, followed by resumption of normoxia (to mimic reperfusion therapy) and electromechanical stimulation for 2 days. Mechanical stimulation was performed at 80 bpm, the normal heart rate, and electromechanical stimulation was performed using the C-PACE EP system, see details in Table 1. Cells in normal conditions were cultured under normoxia throughout and did not have an interruption in electromechanical stimulation.

The heart attack culture model significantly reduced the viability of hiPSC-CMs, HCFs, and HCAECs by 19, 15, and ≈10%, respectively, compared to normal culture (Figure 2B). Notably, the spindle morphology of cardiomyocytes was maintained in the control group but was lost in the heart attack group. RT-qPCR analysis of the cardiac (TNNI3) and skeletal (TNNI1) isoforms of troponin I in hiPSC-CMs revealed a significant 23% decrease in TNNI3 mRNA expression in the heart attack group compared to control and no difference in TNNI1 expression between groups (Figure 2C). RT-qPCR analysis of HCFs showed that MMP2, MMP9, and inhibitors TIMP1, TIMP2 were significantly increased by ≈ 2-, 18-, 20-, and 3-fold, respectively in the heart attack group. No significant differences in the mRNA expression of various markers were observed between the 2 groups of cultured HCAECs.

Our data shows decreased gene expression of myocardial maturation markers in hiPSC-CMs under our electromechanical stimulation model of a heart attack. There have been many reports that electrical and mechanical stimuli play an important role in cardiomyocyte maturation (Brown, 2000; Davis et al., 2015), including our previous work (Cortes et al., 2020). However, to our knowledge, our study is the first to report observations of cell behaviour under transient hypoxia and after interruption of the electromechanical stimuli. It should be noted, however, that the maturation markers used in this study do not fully reflect the hiPSC-CM phenotype; a multifaceted evaluation of gene expression, such as myosin heavy chain 7 (Myh7) and sarcoplasmic reticulum/endoplasmic reticulum Ca2+-ATPase (SERCA), along with an assessment of electrophysiological function (using patch-clamp tests), contractile function, and morphological characteristics is necessary to more conclusively determine their state of maturation (Sacks et al., 2018). The reduced viability of the hiPSC-CMs in the heart attack culture condition was similar to previous reports on the behaviour of hiPSC-CMs under hypoxia (Häkli et al., 2021). Immature myocardium is known to be more tolerant to hypoxia because the glycolytic flux in this state is more active (Patte et al., 2010; Neary et al., 2014). Under hypoxic conditions, myocardial metabolism depends on glycolysis and requires increased glucose uptake and expression of glucose transporters (Rosano et al., 2008). Although these results require careful evaluation of viability, it is possible that this less efficient method of energy production may have contributed to the reduced viability.

It has been reported that MMP-9 activity in cardiac fibroblasts is decreased by hypoxia, a finding also observed in the present study (Riches et al., 2009). MMP-2 and MMP-9 process many collagens, including collagen types I, IV, and V, while MMP-2 also cleaves collagen type III (Steffensen et al., 1995). The proteolytic activity of MMPs is suppressed by TIMPs, the major inhibitors of MMPs in the myocardium (Heymans et al., 2005). Of the four TIMPs identified, TIMP1 levels has been reported to be elevated in hearts post myocardial infarction while TIMP2 was found to be elevated in non-infarcted hearts, respectively (Li et al., 2001; Nuttall et al., 2004; Kandal et al., 2010). TIMP1 and TIMP2 levels were both increased in human cardiac fibroblasts subjected to the heart attack culture protocol in the present study.

Following a myocardial infarction, the endothelial cell survival is a crucial factor in the recovery process (Bauersachs et al., 1999). These cells demonstrate resilience as they adapt to the altered microenvironment caused by the cardiac damage. Previous studies have found that endothelial cells undergo a transient mesenchymal activation within the first days after myocardial infarction but do not acquire a long-term mesenchymal fate (Tombor et al., 2021). However, it remains unclear whether death of endothelial cells is a direct cause of myocardial infarction, or a consequence of disease-induced injury. Our results indicate a reduction in cell viability in HCAECs cultured under mimetic heart attack conditions.

A possible future application for this device in the study of human cardiac fibroblasts is the evaluation of their electrical interactions with cardiomyocytes. Cardiac fibroblasts form functional syncytium by expressing intercellular channels (gap junctions) at sites of cell-cell contact (Goldsmith et al., 2004; Langevin et al., 2004). Cardiac fibroblasts have been reported to respond to mechanical stress by fluctuating membrane potential in synchrony with the contraction of adjacent cardiomyocytes (Kamkin et al., 1999). However, the precise role of these membrane potential fluctuations remains unclear. A previous study has reported that myofibroblasts, which are attached to cardiomyocytes by adhesive junctions, alter the electrophysiology of cardiomyocytes by applying mechanical stress to them and activating mechanosensitive channels, resulting in arrhythmogenic slow conduction (Thompson et al., 2011). This observation proposes an interesting variation of electromechanical interactions, as it does not depend on heterogeneous cell-gap junctions between myofibroblasts and cardiomyocytes. However, it is not yet known if, and to what extent, these variations between fibroblasts and cardiomyocytes result in arrhythmogenic consequences. Co-culturing fibroblasts and cardiomyocytes under the control of this device may provide a means to elucidate some of the mechanisms involved.

## 4 Conclusion

BEaTS-β can be used as an *in vitro* model of cardiac diseases, including acute myocardial infarction and reperfusion injury. Notably, BEaTS-β can mimic the abnormal frequency and intensity of electromechanical stimulation that occurs during adverse cardiac events. In addition, the oxygen concentration and the composition of the cell culture medium can be modulated using commercially available cell incubators. These features enable *in vitro* recapitulation of a wide range of cardiac diseases, including myocardial infarction, tachyarrhythmia, and bradyarrhythmia, as well as normal myocardium. In the future, this device is expected to play a role in the observation of more dynamic cell behavior under abnormal conditions, not only by culturing single cell groups but also by co-culturing multiple cell types. Furthermore, the open-source nature of BEaTS-β will allow for other researchers to expand future applications for *in vitro* studies requiring tunable electromechanical stimulation.

## Data Availability

The datasets presented in this study can be found in online repositories. The names of the repository/repositories and accession number(s) can be found in the article/Supplementary Material.
