# Supplementary material for: BEaTS-β: an open-source electromechanical bioreactor for simulating human cardiac disease conditions
Source: Front Bioeng Biotechnol. 2023 Sep 15;11:1253602. doi: 10.3389/fbioe.2023.1253602 (PMC10540188; doi:10.3389/fbioe.2023.1253602)
Supplement: Supplementary file 1 [file DataSheet1.PDF]

Supplementary information for BEaTS-β: An open-source electromechanical bioreactor for simulating human cardiac disease conditions

Hiroki Takaya[1,†], Maxime Comtois-Bona[1,2,†], Ana Spasojevic[1], David Cortes[1,2], Fabio Variola[3], Wenbin Liang[4,5], Marc Ruel[1], Erik J. Suuronen[1,5*], and Emilio I. Alarcon[1,6*]

[1]Division of Cardiac Surgery, University of Ottawa Heart Institute, Ottawa, ON, K1Y4W7, Canada
[2]Biomedical Mechanical Engineering, University of Ottawa, Ottawa, ON, K1N6N5, Canada
[3]Department of Mechanical Engineering, University of Ottawa, Ottawa, ON, K1N 6N5, Canada
[4]Cardiac Electrophysiology Laboratory, University of Ottawa Heart Institute, Ottawa, ON, K1Y4W7, Canada
[5]Department of Cellular and Molecular Medicine, University of Ottawa, Ottawa, ON, K1H8M5, Canada
[6]Biochemistry, Microbiology and Immunology, University of Ottawa, Ottawa, ON, K1H8M5, Canada
*email: ealarcon@ottawaheart.ca & esuuronen@ottawaheart.ca
[†]These authors contributed equally to this work

Table S1. List of 3D printing parts.

Part name	Quantity	Filament	Printing profile
Plate holder	1	Nylon 680	A
Seal mold-extruded	1	PLA	B
Seal mold-hole	1	PLA	B
Membrane holder_bottom	1*	TPE 80A	C
Membrane holder_top	1*	TPE 80A	C
BEaTS-Beta_casing_bottom	1	ABS	D
BEaTS-Beta_casing_lid	1	ABS	D
BEaTS-Beta_casing_leg	4	ABS	D

Table S2. 3D printing profile for the 3D-printed components.

**Profile A - Nylon 680**	**Profile B - PLA**
o Layer height: 0.2 mm<br>o Wall thickness: 2.0<br>o Top/Bottom thickness: 2.5<br>o Infill density: 35%<br>o Infill pattern: triangles<br>o Printing temperature: 255ºC<br>o Build plate temperature: 60ºC<br>o Print speed: 70 mm/s<br>o Travel jerk: 50 mm/s<br>o Support: Touching build plate (overhang angle 60º)	o Layer height: 0.15 mm<br>o Wall thickness: 1.2 mm<br>o Top/Bottom thickness: 1.2 mm<br>o Infill density: 20%<br>o Infill pattern: triangles<br>o Printing temperature: 200ºC<br>o Build plate temperature: 60ºC<br>o Enable retraction<br>o Print speed: 70 mm/s<br>o Travel jerk: 50 mm/s<br>o Support: Everywhere (overhang angle 60º)
**Profile C - TPE 80A**	**Profile D - ABS**
o Layer height: 0.15 mm<br>o Wall thickness: 0.8 mm<br>o Top/Bottom thickness: 0.8 mm<br>o Infill density: 40%<br>o Infill pattern: Concentric<br>o Printing temperature: 235 ºC<br>o Build plate temperature: 80ºC<br>o Do not enable retraction<br>o Print speed: 15 mm/s<br>o Travel jerk: 30 mm/s<br>o Support: Everywhere (overhang angle 50º)	o Layer height: 0.2 mm<br>o Wall thickness: 1.0 mm<br>o Top/Bottom thickness: 1.0 mm<br>o Infill density: 20%<br>o Infill pattern: Triangles<br>o Printing temperature: 250 ºC<br>o Build plate temperature: 85ºC<br>o Enable retraction<br>o Print speed: 60 mm/s<br>o Travel jerk: 50 mm/s<br>o Support: Everywhere (overhang angle 50º)

Membrane holder

Materials:

- o Membrane holder bottom: 1x each
- o Membrane holder top: 1x each
- o Translucent silicone membrane, 0.5 mm thickness (GraceBiolabs)
- o RTV 108 medical-grade silicone
- o 20 mm histology metal punch
- o 3 mm histology metal punch
- o Torch (handheld/baking)
- o Tape
- o 1x Phosphate Buffer Solution (PBS)
- o 70% ethanol

Assembly protocol

The following steps must be followed to assemble each membrane holder. The number of membrane holders to be assembled will depend on the total number of cells and wells that are needed for the experiment. Each membrane holder replaces one well of a 6-well plate. Cells that are to be mechanically stimulated (and any other combination that includes mechanical stimulation) must be seeded and cultured in modified wells. Control studies can also be conducted seeding cells in the membranes even if the cells are not to be mechanically stimulated (no stimulation or electrical stimulation as well). Both top and bottom membrane holders must be 3D printed prior to the assembly of the holders.

1. Unpack one 6-well plate and put it flat on a solid surface with good ventilation (fume hood is recommended) and no volatile materials nearby.
2. Using a marker or pen, mark the outer circumference of the 20 mm histology punch on the bottom of each well that is to be modified.
3. Using appropriate protective equipment (insulated gloves, goggles, lab coat), use the torch to heat the cutting edge of the metal histology punch.
4. While hot, use the punch to remove the marked circle of the wells.
5. Repeat steps 2-4 for as many wells and well plates as required.
6. While working in a sterile environment (cell culture hood), use the bottom part of the membrane holders to cut squares off the silicone sheet (approx. 25.4 mm length). Each membrane holder needs one square.
7. Using the top membrane holder, cut circles off the tape with the same circumference as the inner circle of the membrane holder. Each membrane holder requires two circles (see **Figure S1B(1)**).
8. Place one circle in the middle of the silicone square, and the other on the opposite side of the membrane.
9. Using the RTV 108 medical-grade silicone, cover the inside of the bottom membrane holder, making sure it is even and covers all the inside.
10. Carefully place the silicone sheet over the membrane holder, making sure all the edges are aligned (see **Figure S1B(2)**).
11. Using the 3 mm punch, lightly press over the pins that are on the bottom holder until little circles are cut off the silicone sheet; be careful not to press too hard to not cut the membrane holder.
12. Allow this subassembly to dry for at least 30 minutes. In the meantime, other membrane holders can be assembled and brought to this step.
13. Insert the top membrane holder through the circular hole that was cut off the bottom of the wells. The top of the membrane holder has a bigger diameter than the hole, but the

neck of this part has the same diameter. Therefore, inserting the holder will find some restriction but once inserted it will create a good seal between the plate and the membrane holder (see **Figure S1C(1)**).

14. Carefully, apply more RTV silicone on the borders of the silicone sheet (the surface that will interface with the top membrane holder). Carefully spread it evenly while trying to avoid putting silicone over the circular tape.
15. Place the bottom subassembly against the top membrane holder, ensuring the edges are aligned. Press lightly the two parts of the membrane holder to remove any air bubbles in the silicone (see **Figure S1C(2-3)**).
16. Add another layer of RTV silicone all around the edges of the now assembled membrane holder, spread it evenly making sure there are no gaps or air bubbles.
17. Allow the silicone to cure for a minimum of 2 hours, a longer curation is suggested for better results (8 – 24h).
18. Once the silicone has solidified, carefully remove the tape from the membrane.
19. In a sterile area (cell hood), rinse each well at least 4x with 70% ethanol. Between rinsing cycles, irradiate the plate under UV light.
20. Rinse the wells at least 4x with 1x PBS.
21. Sterilize the plate under UV light for a minimum of 60 minutes.

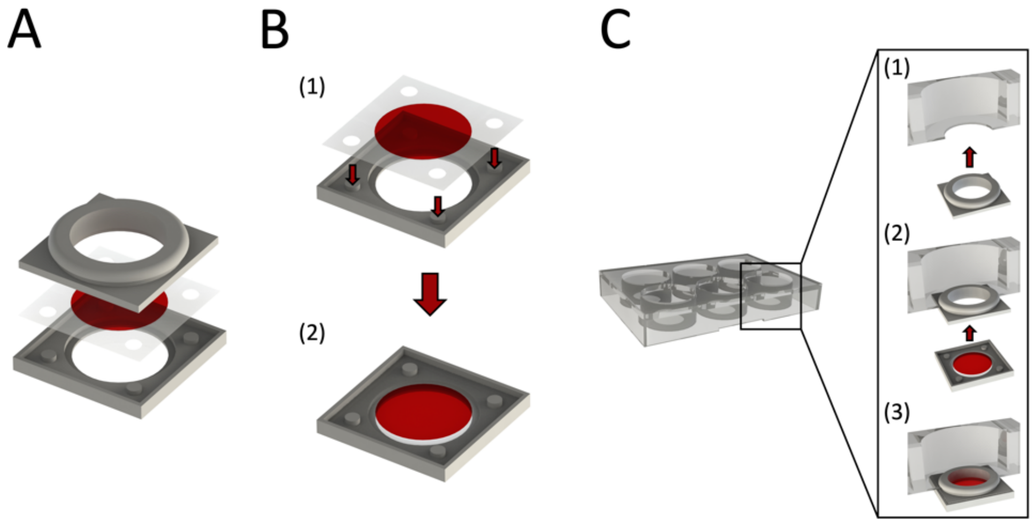

Figure S1. Membrane holder of the BEaTS-β. A) Exploded view of the holder showing the top, and bottom parts, and the silicone sheet. B) Steps of the assembly of the holders. C) Installation of the membrane holder on the modified 6 well-plate.

Full System Assembly

Before the assembling of the stimulation device, all 3D-printed parts must have been printed and cleaned. Each device holds one 6-well plate and uses one electrode plate from the C-PACE EP system.

Materials:

- Membrane holders' subassembly (minimum 1, maximum 6 per well plate, for as many plates as needed)
- Seal mold extruded and Seal mold hole
- RTV 108 medical-grade silicone
- 70% ethanol
- Cell hood with UV light
- 1x Phosphate buffer solution (PBS)
- Laboratory tubbing
- Air pump (capable of producing 55 kPa of pressure)
- Arduino circuit
- C-PACE EP system

Assembly protocol

Plate seal

1. Using the Seal mold extruded and Seal mold holes parts, create the silicone seal.
2. Insert both parts to create a tray where the silicone will be added (see **Figure S2A**).
3. Add the RTV silicone into the mold ensuring that no air bubbles are left and spread it across the mold to ensure the molded silicone is even (see **Figure S2B**).
4. Allow the silicone to cure for a minimum of 24 hours.
5. Once solidified, carefully disassemble the mold and remove the seal.

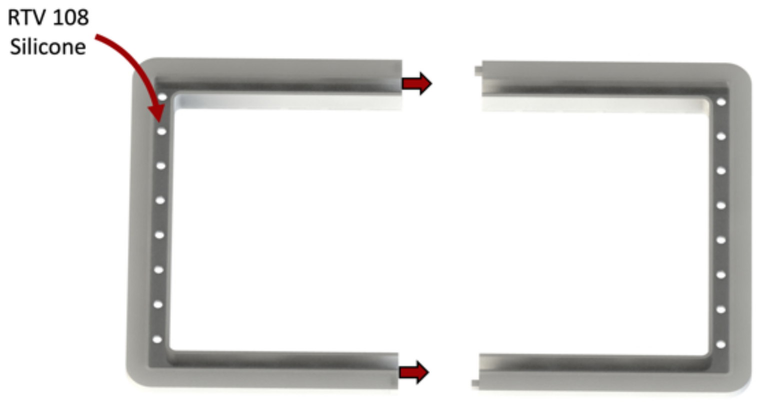

Figure S2. Assembly of the mold to generate the silicone seal.

Plate holder

1. Place the modified 6-well plate on the plate holder (see **Figure 1C from article**).
2. Place the silicone seal around the plate ensuring it fits tightly and creates a good seal (see **Figure 1C from article**).

3. Once the cells are ready to be stimulated, place the C-PACE EP system on the well plate. Make sure the carbon electrodes have been properly cleaned following the manufacturer's protocol.

4. Insert a long strip of laboratory tubbing to both valve heads that extrude from the plate holder (see **Figure 1A from article**). One valve will be connected at the other end to the air pump, and the other will be connected to the Solenoid valve.

    a. Note that both pump and solenoid valve are placed outside of the incubator, to maintain the airflow through the device but never released within the device to prevent contamination, as well as to eliminate the placement of electrical components within the incubator to reduce the risk of corrosion, overheating, and contamination.

5. Using the cabling provided by the manufacturer, connect the C-PACE EP plate to the control unit.

Casing – control unit

- o Following the circuit connections shown in **Figure S3**, see below, the control unit must be placed within the 3D printed casing as follows.
- o Place the Arduino UNO, breadboard, relay module, solenoid valve, 12V port, and BNC connector in each compartment of the casing.
- o Connect all the components following the circuit provided, note that the casing contains holes so that the jumper wires can be passed through, to maintain a cleaner and more organized cabling within the device.
- o Once all components have been placed within the casing and the connections are finished, the lid (not shown) can be placed on top of the casing.
- o Once located, the three pushbuttons can be inserted in their respective places (holes found on the lid), as well as the LED bulbs.
- o Note, the colour of the buttons and LEDs can be interchanged if desired. It is recommended to keep each pair of button:LED with the same colour, and having 3 colours, each corresponding the one stimulation mode (normal heartbeat, arrhythmia, and heart failure).
- o Once assemble, connect the laboratory tubbing to one of the ports of the solenoid valve.
- o Similarly, connect the 12V power supply to the 12V port, and the BNC connector from the C-PACE EP system to the BNC connector outlet on the casing.

Table S3. Components of the BEaTS-β Arduino controller.

Part name	Model, Brand	Quantity
Arduino Microcontroller	Arduino UNO R3, Elegoo (or any generic brand)	1
Solenoid valve	TRTV1555 Solenoid Valve, Yooso	1
Relay module	JQC-3FF-S-Z 5V relay, Tongling	1
Pneumatic Quick Connect Fittings (for Solenoid Valve)	5779K109 Push-to-Connect Tube Fitting for Air, McMaster-Carr	
Pushbuttons	12mm Push Button Momentary Switch, Gikfun	3
12V power supply		1
LED bulbs		3
1 kΩ resistor		6
Male BNC Arduino connector		1
Male-to-male BNC cable		1
Jumper wires		Multiple
Breadboard		1

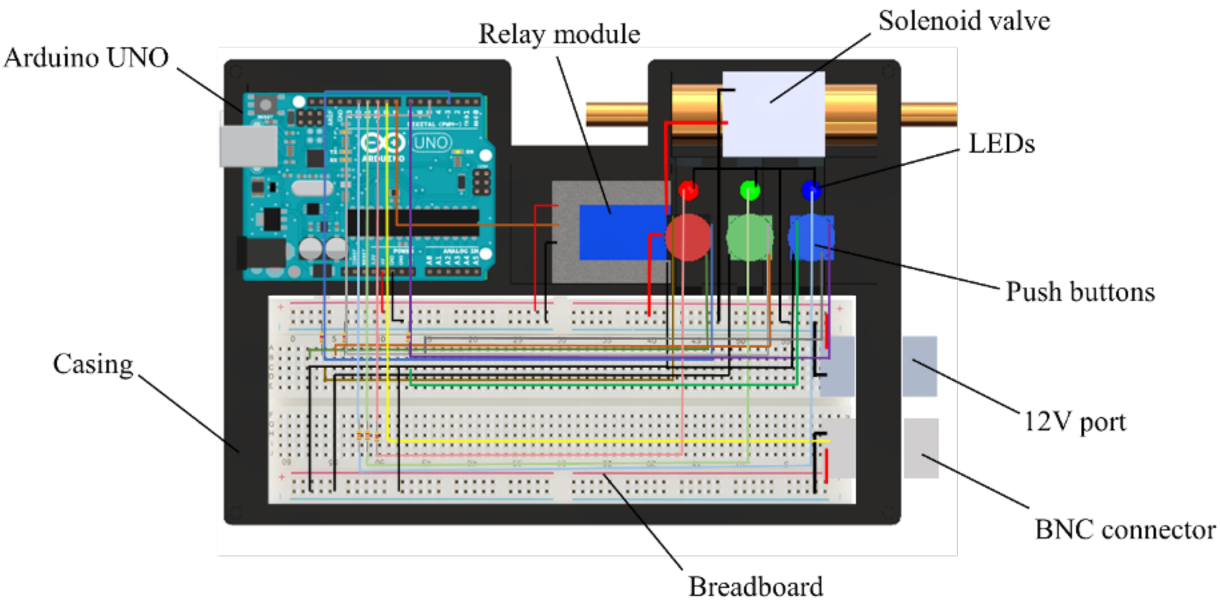

Figure S3. Schematic of the circuit for the BEaTS-β Arduino controller.

Table S4. Code variables, values, and function.

Variable name	Value assigned	Function
Int normal	20	Sets the rate of stimulation **during the normal heartbeat mode.**
Int tachy	10	Sets the rate of stimulation **during the arrhythmia and heart failure modes,** throughout the tachycardia stimulation phase.
Int brady	30	Sets the rate of stimulation **during the arrhythmia and heart failure modes,** throughout the bradycardia stimulation phase.
Int Acount	0	Used to count the ¨beats¨ during the **arrhythmia mode.**
Int Hcount	0	Used to count the ¨beats¨ during the **heart failure mode, before and during heart attack simulation.**
Int HFcount	0	Used to count the ¨beats¨ during the **heart failure mode, after heart attack simulation.**

Table S5. Primer sequences used in the qRT-PCR of this study.

Gene	Sense primer	Antisense primer
TNNI1	GACCTCACAATAGAGGCCAGC	GGAAGGGATGCTCCAGACAC
TNNI3	CTTCGAGGCAAGTTTAAGCGG	GGTTTTCCTTCTCGGTGTCCT
MMP2	TACAGGATCATTGGCTACACACC	GGTCACATCGCTCCAGACT
MMP9	GGGACGCAGACATCGTCATC	TCGTCATCGTCGAAATGGGC
TIMP1	AGAGTGTCTGCGGATACTTCC	CCAACAGTGTAGGTCTTGGTG
TIMP2	AAGCGGTCAGTGAGAAGGAAG	GGGGCCGTGTAGATAAACTCTAT
eNOS	GTTTGTCTGCGGCGATGTT	GCGTGAGCCCGAAAATGTC
KLF2	CCACGATCCTCCTTGACGAG	CCGCAGACAGTACAAATTAAGGC
KLF4	CAGCTTCACCTATCCGATCCG	GACTCCCTGCCATAGAGGAGG
ERK5	GCTGGCGCTCCTGGGCTGTCACC	GAGCCTGCCCCACCAAAGAAAGAT
END1	AGAGTGTGTCTACTTCTGCCA	CTTCCAAGTCCATACGGAACAA
ICAM-1	TGCCACCAATATGGGAAGGC	CCGAGCTCAAGTGTCTAAAG
VCAM-1	GGTGCTGCAAGTCAATGAGA	AAGATGGTCGTGATCCTTGG
PPIA	AATGCTGGACCCAACAC	TCCACAATATTCATGCCTT
CCL2	CTCATAGCAGCCACCTTCATT	CACAGCTCCCTTGGCCACAAT
GAPDH	AGCCACATCGCTCAGACAC	GCCCAATACGACCAAATCC
